# Successful twin pregnancy in a patient with parkin-associated autosomal recessive juvenile parkinsonism

**DOI:** 10.1186/1471-2377-11-72

**Published:** 2011-06-17

**Authors:** Takehiro Serikawa, Takayoshi Shimohata, Mami Akashi, Akio Yokoseki, Miwa Tsuchiya, Arika Hasegawa, Kazufumi Haino, Ryoko Koike, Koichi Takakuwa, Keiko Tanaka, Kenichi Tanaka, Masatoyo Nishizawa

**Affiliations:** 1 Department of Obstetrics and Gynecology, Niigata University Medical and Dental Hospital, Niigata 951-8510, Japan; 2 Department of Neurology, Brain Research Institute, Niigata University, Niigata 951-8585, Japan; 3 Department Neurology, Nishi-Niigata Chuo National Hospital, Niigata 950-2085, Japan; 4 Department of Neurology, Kanazawa Medical University, Kanazawa 920-0293, Japan

## Abstract

**Background:**

Pregnancy in patients with Parkinson disease is a rare occurrence. To the best of our knowledge, the effect of pregnancy as well as treatment in genetically confirmed autosomal recessive juvenile parkinsonism (ARJP) has never been reported. Here, we report the first case of pregnancy in a patient with ARJP associated with a *parkin *gene mutation, ARJP/PARK2.

**Case presentation:**

A 27-year-old woman with ARJP/PARK2 was diagnosed as having a spontaneous dichorionic/diamniotic twin pregnancy. Exacerbation of motor disability was noted between ovulation and menstruation before pregnancy as well as during late pregnancy, suggesting that her parkinsonism might have been influenced by fluctuations in the levels of endogenous sex hormones. During the organogenesis period, she was only treated with levodopa/carbidopa, although she continued to receive inpatient hospital care for assistance in the activities of daily living. After the organogenesis period, she was administered sufficient amounts of antiparkinsonian drugs. She delivered healthy male twins, and psychomotor development of both the babies was normal at the age of 2 years.

**Conclusion:**

Pregnancy may worsen the symptoms of ARJP/PARK2, although appropriate treatments with antiparkinsonian drugs and adequate assistance in the activities of daily living might enable successful pregnancy and birth of healthy children.

## Background

Pregnancy in patients with Parkinson disease (PD) is a rare occurrence. However, physicians treating pregnant patients with PD should be aware of the impact of pregnancy on parkinsonism as well as that of the treatment for PD on pregnancy and fetal development [1,2]. To the best of our knowledge, the effect of pregnancy in genetically-confirmed autosomal recessive juvenile parkinsonism (ARJP) has never been reported. Here, we report the first case of pregnancy in a patient with ARJP associated with a *parkin *gene mutation (ARJP/PARK2), which is characterized by early onset, marked response to levodopa treatment, and levodopa-induced dyskinesia [3].

### Case Presentation

A 27-year-old woman with ARJP having heterozygous exonic deletion mutations (Δexon 4/Δexons 2-4) of the *parkin *gene (patient II-2 in pedigree 455) [4] was referred to our hospital at the fifth week of gestation. This patient showed bradykinesia, rigidity, postural instability, dystonia, a slowly progressive course, and alleviation of symptoms in response to levodopa therapy. She had a 15-year history of parkinsonism and was receiving treatment with dopaminergic drugs, including levodopa/carbidopa (450 mg/day), entacapone (400 mg/day), selegiline (7.5 mg/day), and ropinirole (1.5 mg/day). Wearing-off phenomenon and levodopa-induced dyskinesia were noted since the age of 24 years. The wearing-off phenomenon became remarkable gradually. The duration of levodopa action was about 120 min. Treatment with entacapone increased the duration of the "on" period. The Hoehn and Yahr stage was 5 during the "off" period and 2 during the "on" period. Moderate dyskinesia with pain was prominent at the peak dose of levodopa medication. Interestingly, her parkinsonism tended to fluctuate depending on the menstrual cycle since the age of 21 years: it exacerbated between ovulation and menstruation.

She was diagnosed as having a spontaneous dichorionic/diamniotic twin pregnancy. To reduce the teratogenic risk of antiparkinsonian drugs [5], we eventually discontinued them except levodopa/carbidopa (450 mg/day) until the sixth week of gestation. Thereafter, her dyskinesia disappeared almost completely during the course of gestation. Because the patient complained of worsening of her motor disability (Hoehn and Yahr stage [on/off] 3/5) and felt strong anxiety due to her inability to move during off-time and in the night, she received inpatient hospital care for assistance in the activities of daily living (ADL) without increasing the dose of the antiparkinsonian drugs. However, her motor disability worsened after the 19^th ^week of gestation (Hoehn and Yahr stage [on/off] 4/5) and the wearing-off phenomenon became remarkable after the 21^st ^week of gestation. Thus, we gradually increased the dose of levodopa/carbidopa up to 700 mg/day and resumed entacapone and selegiline at doses of 400 mg/day and 7.5 mg/day, respectively.

At the 35^th ^week of gestation, the patient underwent caesarean delivery due to preterm premature rupture of the membranes and onset of labor pains. She delivered healthy male twins weighing 2,184g and 2,022g with 5-min Apgar scores of 8 and 9, respectively. Examination of the babies revealed no abnormalities except ventricular septum defect in one of the babies. Because the diameter of the VSD measured by using transthoracic echocardiography was small (3 mm), it was considered that this VSD did not need surgical repair. The babies were fed synthetic milk to avoid exposure to antiparkinsonian drugs, because medical information on this topic is lacking. After discharge from our hospital, the patient could care for the babies with assistance from her family. Psychomotor development of both the babies was normal at the age of 2 years.

## Conclusions

In this report, we present the case of a twin pregnant patient with ARJP/PARK2. It remains unknown whether the twin pregnancy was associated with the exposure to several antiparkinsonian drugs taken during early pregnancy. Whether the twin pregnancy was associated with the exposure to several antiparkinsonian drugs taken during early pregnancy remains unknown, because a causal association between antiparkinsonian drugs and multiple ovulations has not yet been established.

We also present several important findings in pregnant PD patients. First, we speculated that parkinsonism associated with ARJP/PARK2 might be influenced by the levels of sex hormones. In our patient, exacerbation of motor disability was noted between ovulation and menstruation before pregnancy as well as during late pregnancy. Because estrogen increases during such conditions and because estrogen is known to regulate the dopaminergic neurotransmission in the basal ganglia [6], estrogen may be considered to exert an influence on parkinsonism. However, there is a possibility that the motor disability noted during late pregnancy could be related to pregnancy-induced pharmacokinetic variations of drug levels [6].

Second, programmed pregnancy might be recommended to PD patients to reduce the risk of teratogenicity. We considered that monotherapy with levodopa/carbidopa might be preferable during the organogenesis period, because this drug combination has been shown to have no adverse effect on the fetus [7]. However, because monotherapy with levodopa/carbidopa could increase parkinsonism and the off-time duration, adequate ADL assistance should be provided to the patients during this period. With regard to the presence of a VSD in one of the twins, a potential role of medication effect of antiparkinsonian drugs needs to be considered. However, because this anomaly is not very rare (the prevalence of muscular VSD in neonates is 53.2/1000 live births [8]), it is difficult to conclude that VSD is caused by antiparkinsonian drugs.

Finally, after the organogenesis period, patients should be administered sufficient amounts of antiparkinsonian drugs to improve their parkinsonism symptoms and the wearing-off phenomenon, because a worsened clinical status may have a profound impact on the maintenance of pregnancy, delivery, and the ability of PD patients to care for their babies [9].

In conclusion, pregnancy may worsen the symptoms of ARJP/PARK2, although appropriate treatments with antiparkinson drugs and adequate ADL assistance might enable successful pregnancy and birth of healthy children.

## Abbreviations

PD: Parkinson disease; ARJP: autosomal recessive juvenile parkinsonism

## Competing interests

The authors declare that they have no competing interests.

## Authors' contributions

TS and TS drafted the first manuscript and made a contribution to acquisition and interpretation of data. MA, MT, and KH are treating obstetricians of the patient, and made a contribution to literature search. AY is a treating neurologist of the patient during delivery, and made a contribution to literature search. KT and MN revised the manuscript that led to the final approval of the current submission. All authors read and approved the final manuscript.

## Pre-publication history

The pre-publication history for this paper can be accessed here:

http://www.biomedcentral.com/1471-2377/11/72/prepub
